# CRISPR/Cas9 and cancer targets: future possibilities and present challenges

**DOI:** 10.18632/oncotarget.7104

**Published:** 2016-01-31

**Authors:** Martyn K. White, Kamel Khalili

**Affiliations:** ^1^ Department of Neuroscience, Center for Neurovirology and Comprehensive Neuroaids Center, Temple University School of Medicine, Philadelphia, PA, USA

**Keywords:** CRISPR/Cas9, cancer genome manipulation, oncogene disruption, gene correction, gene therapy

## Abstract

All cancers have multiple mutations that can largely be grouped into certain classes depending on the function of the gene in which they lie and these include oncogenic changes that enhance cellular proliferation, loss of function of tumor suppressors that regulate cell growth potential and induction of metabolic enzymes that confer resistance to chemotherapeutic agents. Thus the ability to correct such mutations is an important goal in cancer treatment. Recent research has led to the developments of reagents which specifically target nucleotide sequences within the cellular genome and these have a huge potential for expanding our anticancer armamentarium. One such a reagent is the clustered regulatory interspaced short palindromic repeat (CRISPR)-associated 9 (Cas9) system, a powerful, highly specific and adaptable tool that provides unparalleled control for editing the cellular genome. In this short review, we discuss the potential of CRISPR/Cas9 against human cancers and the current difficulties in translating this for novel therapeutic approaches.

## INTRODUCTION

Since all cancers contain multiple mutations that allow them to grow progressively and exhibit the characteristics of malignancy [1, 2], targeting the cancer cell genome is an attractive approach. These mutations fall into a number of categories that confer distinct biological capabilities and are acquired during the multistep process of tumor development. These characteristics, often called hallmarks of cancer [1, 2], include activating and sustaining the signaling processes necessary for cell proliferation, evading the normal function of growth suppressors, invasion and metastasis as more fully elaborated below. The ability to correct such cancer-associated mutations is an attractive approach as a treatment option. Such a treatment requires a reagent which should induce the correcting genetic changes in a highly specific manner with limited off-target effects. The reagent would require efficient delivery into all or nearly all of the cells in a tumor in order to be effective. In this review, we discuss the recently developed CRISPR/Cas9 system and its potential in this regard.

## CANCER

It is an important and well-established that cancer is a genetic disease caused by mutations in the cellular genome that are usually somatic in nature. The development of tumors is a multistep process in which several mutations are required and each mutation contributes to deregulation of cellular proliferation associated with a gradual increase in the size of the tumor, its level of disorganization and malignant potential with at least three to six mutations being to be required for full malignancy to be realized [3]. The classic paradigm for this multistep process is the adenoma—carcinoma sequence of colorectal neoplasia described by the Vogelstein laboratory [4]. In addition to the mutations described in the landmark studies from the Vogelstein group, e.g., p53, pRb, DCC, APC, etc., many novel oncogenic mutations continue to be discovered especially with the development of powerful techniques that facilitate large-scale genomic studies, which have revealed a plethora of new oncogenes, including many in processes that were not previously known to be involved in cancer [5, 6]. While some of these mutations are thought to arise as a result of environmental factors such as chemical mutagens, it is possible that many are due to random mutations arising during DNA replication in normal, noncancerous stem cells [7]. It is also important to note that about a fifth of all human cancers are caused by infectious agents, especially viruses [8. 9]. It is of note that the many pathways that are affected by oncoviruses to establish tumors are relevant to the hallmark characteristics of cancer discussed below.

The common characteristics of cancers arising during the multistep mutational process of carcinogenesis can be designated into hallmarks, which constitute an organizing principle for rationalizing the complexities of neoplastic disease [1, 2]. Possibly the most basic trait of tumor cells is their ability to activate and sustain continuous proliferation. Thus mutations in the pathways of growth factors that bind and activate cell-surface receptors, often with intracellular tyrosine kinase domains are common in cancer. This group includes the viral and cellular oncogenes. Similarly, disruptions of negative-feedback mechanisms that attenuate proliferative signaling constitute a second hallmark group of cancer mutations, e.g., mutational inactivation of tumor suppressor genes. Other important hallmarks are resisting apoptotic or senescent cell death, acquisition of replicative immortality, reprogramming of cellular energy metabolic pathway utilization, evading destruction by the immune system, the ability to induce angiogenesis, and activating invasion and metastasis.

Another feature of many cancer cells is a so-called mutator phenotype, which may facilitate the occurrence of genetic changes. This may involve changes in the level or mutation of enzymes involved in DNA modification or repair [10]. Also of importance are changes that enable cells to escape the effects of toxic chemotherapeutic drugs, i.e., chemoresistance and such changes include enhanced expression of ABC transporter proteins leading to drug efflux and changes in the levels of enzymes responsible for drug activation or inactivation [11].

Since all cancers have multiple mutations, this raises the possibility that correcting or ablating one or more sections of the genome may provide a potent approach against cancer.

## GENOME MANIPULATION TOOLS

In the last several years, novel genetic-engineering technologies have been developed enabling precise editing of genomes and these have a number of important clinical applications including the treatment of genetic diseases, viral infections and cancer. These new classes of reagents, which can specifically target nucleotide sequences within cellular genomes, are of three major types. Firstly, there are the zinc-finger nucleases (ZFN), which are fusion proteins consisting of the enzymatic cleavage domain of the FokI restriction endonuclease with custom-designed Cys2-His2 zinc-finger proteins, which confers the specificity [12–14]. Secondly, there are the transcription activator-like effector nuclease (TALEN) system, which are also FokI fusion proteins but the sequence-specific DNA-binding the targeting domain is derived from the transcription activator-like effector (TALE) proteins secreted by Xanthomonas bacteria [15–17]. Thirdly, there is the clustered regulatory interspaced short palindromic repeat (CRISPR)-associated 9 (Cas9) system, which is the most powerful and versatile and provides exceptional control over genome editing [18–24]. This has the most potential to engineer cancer cells and we will concentrate on this approach in this review.

**Figure 1:**
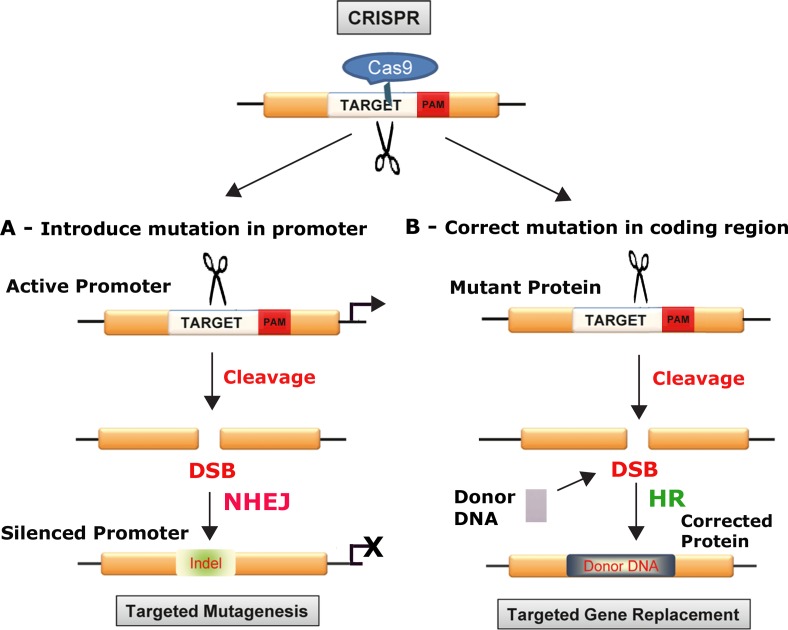
Schematic of gene disruption (A) and correction (B) approaches with CRISPR/Cas9: In the top panel, the relationship of the target DNA sequence, the Protospacer Adjacent Motif (PAM) trinucleotide sequence and Cas9 protein to the scission of the target Cas9 cuts both strands of DNA causing a DSB, which lies 3-4 nucleotides upstream of the PAM sequence, which can be used to either disrupt DNA by targeted mutagenesis (A) or replace and correct a mutated gene as shown below (B). CRISPR/Cas9 can be used to silence a promoter. **A.** double-stranded DNA break is introduced by specific cleavage and this is repaired by the error-prone process of nonhomologous end-joining DNA repair (NHEJ), which introduces InDel mutations that can disrupt the function of a promoter region. This method can be used to disrupt oncogenes, e.g., myc that is expressed at a high level due to translocation into immunoglobulin loci in Burkitt's lymphomas. **B.** CRISPR/Cas9 can be used to repair and correct a mutated gene. Double-strand DNA breaks are introduced by specific cleavage but in this case repair is mediated by the high-fidelity mechanism of homologous recombination-directed DNA repair (HR). This can be used to repair mutated tumor suppressor genes and restore the wild-type sequence and function.

CRISPR is a system of prokaryotic adaptive immunity from which the CRISPR/Cas9 technology emerged [18, 20, 22]. The CRISPR/Cas system is found in ~90% of archaea and ~50% of bacteria and evolved as a defense against viruses [25]. From early in 2013 onwards, many studies have demonstrated that site-specific editing of DNA in eukaryotic cells can be achieve by co-expressing the Cas9 enzyme from Streptococcus pyogenes and a short guide RNA (gRNA). The power of the CRISPR/Cas9 system lies in its simplicity, ease of use, adaptabilty and flexibility to different targets [26]. Cas9 is an endonuclease that targets specific DNA sequences through Watson-Crick DNA:RNA base pairing to the gRNA so that changing the sequence of the RNA is an easy way to change the DNA specificity [27]. The gRNA is designed to contain a 20 base-pair guide sequence, which recruits the Cas9/gRNA complex to its target and also contains a sequence known as the Protospacer Adjacent Motif (PAM) trinucleotide sequence immediately following the target sequence. Cas9 cuts both strands of DNA causing a double-strand break (DSB), at a point which lies 3-4 nucleotides upstream of the PAM. DSBs are repaired by the non-homologous end-joining (NHEJ) DNA repair pathway. Since NHEJ is error-prone, it frequently results in the generation of insertions/deletions (InDels) at the DSB site and this can lead to frameshifts or premature stop codons, which will disrupt the open reading frame (ORF) of the target gene (Figure 1). More precise genome editing can be achieved using homology-directed DNA repair (HDR), by including a homologous donor DNA, which provides a template to introduce new sequences into the gene of interest, e.g., installation of new specific mutations (Figure 1).

Cas9 is a general endonuclease with no intrinsic sequence specificity, so that small gRNA can simply be produced by chemically synthesis, in vitro transcription or by expression in the cell. This straightforwardness of application has allowed its use in thousands of applications over the last two and a half years [18, 20, 22, 23]. Another advantage of the CRISPR/Cas9 system is that it is easy to achieve multiplex targeting by the use of multiple gRNAs. For example, using two gRNAs specific for sites that flank a gene of interest can be used to make chromosomal deletions. In addition to being versatile and simple to use, CRISPR/Cas9 is notable in that it has a high degree of specificity with regard to almost exclusive on-target cleavage, which is very important given the large size of the human genome. To assess off-target cleavage, two approaches are possible: the SURVEYOR assay, which detects mismatched nucleotide pairs resulting from NHEJ, and whole genome sequencing. SURVEYOR assays or whole-genome sequencing at high coverage has revealed the to assess off-target cleavage to be a very rare event [28–31] and the Cas9 system has thus been shown to be highly specific.

**Table 1:** Potential strategies for CRISPR/Cas9 interventions targeting cellular genes in cancer

Gene Type	Family Example	Gene	Function	Approach	Gene Reference
*Oncogene*	Receptor tyrosine kinase	ErbB	Tyrosine kinase	Disrupt or mutate	48, 58
	Nonreceptor	src, abl, fps, yes,	Tyrosine kinase	Disrupt or mutate	49
	G proteins	ras	Molecular Switch	Disrupt or mutate	50
	Signaling kinase	raf	Serine/threonine kinase	Disrupt or mutate	51
	Transcription regulator	myc	Transcription factor	Disrupt or mutate	52
*Tumor Suppressor*	Pocket protein	pRb	Cell cycle regulator	Repair	78-80
	Gene Regulation	p53	Transcription	Repair	81
	Signal regulation	PTEN	Protein phosphatase	Repair	82
	DNA repair	BRCA1/2	DNA repair	Repair	83, 84
	DNA damage checkpoint	ATM	Serine/threonine kinase	Repair	85
*Epigenetic Conrol loci*	DNMTs	DNMT1	DNA methyltransferase	Repair	101
	Histone modification	EZH2/	Histone methylase demethylase	Repair	103, 104
*Chemo-resistance*	Efflux pumps	MDR-1 MRP	ABC transporter	Repair	107, 108
	Drug metabolism	GST-p	GSH conjugation	Repair	111
		Cytokine P450	Inactivation	Repair	112
		UGT1A1	Gluconylation	Repair	113

CRISPR/Cas9 is still a relatively new technology and it continues to be modified and improved for increased editing efficiency and decreased potential off-target events. One such approach is the “paired Cas9 nickase” strategy, which increases site specificity for the induction of DSBs with much reduced off-target occurrences [32–35]. Another approach is to construct a fusion protein of a catalytically dead Cas9 to the FokI restriction endonuclease to generate a highly specific RNA-guided nuclease [36–38]. Inactivating the two catalytic active sites of Cas9 endonuclease by mutation results in an inactive form, which can still bind to target DNA in a gRNA-dependent manner. Bound inactive Cas9 can negatively regulate expression of a gene by sterically blocking access of RNA polymerase to the promoter of the gene [39]. More precise and complex control of gene expression can be achieved by fusing the inactive Cas9 to transcriptional activator or repressor domains [40–43] and indeed more complex approaches have allowed the engineering of synthetic transcriptional programs [44].

Finally, one problem with the Cas9 system is that the large size of the protein limits the vectors that can be used for delivery, as discussed below. One solution has been to look for proteins with a similar function but a smaller size. Ran et al [45] characterized six smaller Cas9 orthologues and found that Cas9 from Staphylococcus aureus (SaCas9) can edit the genome with efficiencies similar to those of the usual Cas9 from Streptococcus pyogenes, while being more than 1 kilobase shorter. Another possibility is to split the Cas9 enzyme and deliver the halves separately. Wright et al [46] designed a split-Cas9 enzyme where the nuclease lobe of Cas9 and the α-helical lobe of Cas9 are expressed as separate polypeptides. The sgRNA recruits the halves into a ternary complex which recapitulates the activity of full-length Cas9 and catalyzes site-specific DNA cleavage [46]. Similarly, Truong et al [47] developed a split-Cas9 using split-inteins and intein-mediated trans-splicing reconstituted the full-length Cas9 protein.

## TARGETS FOR GENOME MANIPULATION TOOLS IN CANCER CELLS

As described above, cancer cells are characterized by the stepwise acquisition of multiple mutations during their development and these mutations can be classified into a number of different groups depending on the function of the gene. These include activated oncogenes, inactivated tumor suppressors, mutations in epigenetic factors and their control loci, mutations in genes that confer chemoresistance genes and others. We will consider each group and how they may serve as suitable targets.

### Oncogenes

The classic oncogenes are drivers of the pathways that control cellular mitogenesis and were first characterized in the 1970s onwards. Examples are receptor tyrosine kinases, e.g., ErbB, derived from the epidermal growth factor receptor [48], which is overexpressed in many cancers especially breast cancer and is targeted by the drug Herceptin, and src-like non-receptor tyrosine kinases, e.g., src, which is mutated in many cases of cancer of the colon, liver, lung, breast and the pancreas [49]. Other classes of oncogene include the G-protein family, e.g., Ras, which are very common, being found in 20-30% of all human tumors [50] and intracellular protein kinases, e.g., Raf, with 20% of all human tumor displaying a mutated B-Raf gene, which has been targeted in cases of renal cell carcinoma and melanoma [51]. Nuclear transcription factors can also be oncogenes, e.g., myc, which is amplified in a significant number of epithelial ovarian cancers and also in breast, colorectal, pancreatic, gastric and uterine cancers [52]. The generation of an oncogene from a normal cellular gene (proto-oncogene) may occur by mutation causing an increase in protein (enzyme) activity or a loss of normal regulation, by an increase in protein expression level or a chromosomal translocation event leading to a fusion to a second gene, e.g., the Philadelphia Chromosome, which creates bcr-abl and is most commonly associated with chronic myelogenous leukemia (CML) [53]. The discovery of new oncogenes continues apace with several examples in the last year, e.g., collagen triple helix repeat containing 1 (CTHRC1) [54], Stratifin (SFN) [55] and Lin28B [56]. The development of powerful techniques for large-scale genomic analysis has resulted in the discovery of many novel oncogenic mutations and a multitude of new oncogenes, including many in processes that were not previously known to be associated with cancer [5, 6]. Indeed, a fundamental problem with cancer genome studies has become that the list of putatively significant genes has burgeoned into the hundreds and novel analytical methodology has had to be developed to eliminate apparent artefactual findings and enable identification of genes truly associated with cancer [57].

Oncogenes drive cell proliferation by a gain-of-function ability to stimulate cell signaling pathways inappropriately. Since they are usually active in the presence of a wild-type allele of the proto-oncogene, they can be said to act in a dominant fashion. Inactivation of an oncogene by a genetic reagent such as CRISPR/Cas9 could be achieved by disrupting a protein motif that is necessary for the activity of the oncoprotein. For example, the src family of oncogenes requires tyrosine kinase activity to transform and could be targeted by CRISPR/Cas9 directed towards the tyrosine kinase domain [58].

As noted above, perhaps as many as a fifth of all human cancers are caused by viruses [8, 9] and some of these encode viral oncogenes that promote carcinogenesis. Hepatitis B virus (HBV), which causes acute and chronic liver infections can lead to hepatocellular carcinoma (HCC). HBV is a hepadnavirus with a small, circular, partially double-stranded DNA genome [59]. The genome of HBV is small and encodes several proteins including HBV Protein X (HBx) and HBV surface antigen (HBsAg), which are important in transformation but not well understood. HBx is oncogenic and can transform rodent hepatocytes and NIH 3T3 cells [60] and may act by activating signal transduction pathways that regulate of cell proliferation such as ERKs, SAPKs and p38 protein kinase [61]. Chronic hepatitis B involves episomal persistence of HBV DNA known as covalently closed circular DNA (cccDNA), and thus curing chronic HBV infection will require the specific eradication of the persistent HBV cccDNA from infected cells. The CRISPR/Cas9 system is a good candidate for this treatment. In this regard, Lin et al [62] tested eight gRNAs against HBV and found that CRISPR/Cas9 was able to considerably reduce the levels of HBV core and HBsAg proteins in Huh-7 hepatocyte-derived cellular carcinoma cell cells transfected with an HBV-expression vector. Furthermore, cleavage of intrahepatic HBV genome-containing plasmids was achieved resulting in viral clearance in an in vivo mouse model, which also showed reduced in serum HBsAg [62]. Seeger and Sohn [63] tested HepG2 hepatoma cells expressing HBV receptor with HBV-specific gRNAs and found inhibition of HBV infections up to eightfold by CRISPR/Cas9 cleavage. Kennedy et al [64] used lentiviral transduction of Cas9 and HBV-specific gRNAs to inhibit HBV DNA production for in vitro models. Zhen et al [65] targeted the HBsAg and HBx in a cell culture system and in vivo, resulting in reduction of HBsAg levels in cultures media sera of mice respectively. In the last few months, there has been a flurry of similar studies on HBV reporting essentially similar findings [66–70]. In conclusion, studies from several labs show the promise of CRISPR/Cas9 as a potential treatment of HBV-associated HCC.

Other viruses express oncogenes that are associated with human cancer and are potential targets for CRISPR/Cas9. Epstein-Barr virus (EBV): EBV is a herpesvirus that causes Burkitt's lymphoma and nasopharyngeal carcinoma [71]. EBV can enter a latent state in B lymphocytes, where the circular episomal EBV genome expresses proteins that can lead to cellular transformation, e.g., EBV nuclear antigen 1 (EBNA-1)[72], LMP-1 and LMP-2 [73] as well as RNAs such as EBV-encoded small RNA-1 and -2 (EBER1 and EBER2) and the BamHI rightward transcripts (BARTs), which effect cellular transformation by multiple molecular mechanisms [74]. EBV can be targeted by CRISPR/Cas9. Patient-derived cells from a Burkitt's lymphoma showed dramatic proliferation arrest and decrease in viral load with an EBV-specific CRISPR/Cas9 vector [75]. Yuen et al [76] also reported CRISPR/Cas9-mediated editing of EBV in human cells with no off-target cleavage was found by deep sequencing. Kaposi's sarcoma herpesvirus (KSHV) is a double stranded human oncogenic DNA virus that expresses oncogenes that are potential targets for CRISPR/Cas9.

As well as viral oncogenes, cellular genes that are mutated to become oncogenes have a huge potential as targets for treating human cancer. Oncogene changes occur in many cancers and are an important driving force for malignant cell proliferation. CRISPR/Cas9 could be targeted against the mutated form of the cellular oncogene to disrupt and inactivate it. For example, non-receptor tyrosine kinase can be inappropriately activated by mutations in their regulatory domains [58]. These oncogenes could be targeted using CRISPR/Cas9 and gRNA directed against the tyrosine kinase domain, which is necessary for oncogenic activity. The CRISPR/Cas9 system has the potential to be developed to provide a specific and efficacious approach against many types of oncogenic changes in cancer cells.

### Tumor suppressors

At least as important as oncogene activation in carcinogenesis is the inactivation of tumor suppressor genes. The existence of tumor suppressors was first revealed in cell fusion where it was shown that hybrids between malignant and normal cells lost their tumorigenicity [77]. If oncogenes are the drivers of cancer, then tumor suppressors are the brakes. Examples are retinoblastoma protein (pRb), which is mutated not only in retinoblastoma but also in some glioblastomas and adenomas [78–80]. The p53 tumor suppressor is perhaps the most commonly mutated in human cancer with >50% of human tumors containing a p53 mutation or deletion [81]. The phosphatase and tensin homolog tumor suppressor (PTEN) is a negative regulator of certain signaling pathways and is frequently inactivated in glioblastoma, endometrial cancer and prostate cancer [82]. Susceptibility to breast cancer can involve the tumor suppressors breast cancer-1 (BRCA1)[83] and breast cancer-2 (BRCA2), which are also involved in ovarian cancer [84]. The ataxia telangiectasia (ATM) tumor suppressor is involved in the cellular response to DNA damage and is mutated in certain kinds of leukemias and lymphomas [85].

Opposite to oncogenes, tumor suppressors are inactivated during the multistep progression of cancer and this may occur in a number of ways. The gene may become inactivate by a loss-of-function mutation or it might be expressed at a much lower level. Note that, unlike oncogenes, tumor suppressor genes usually follow a “two-hit hypothesis” mode of action, i.e., both alleles of the gene encoding for a tumor suppressor protein must be changed before the function is ablated. If only one allele were damaged, the second still produces functional wild-type protein, i.e., mutant tumor suppressor alleles are recessive while mutant oncogene alleles are dominant. A corollary of this is that corrective application of CRISPR/Cas9 to a mutated tumor suppressor necessitates precise reversion to the wild-type sequence.

The BRCA1 protein product has a role in DNA repair [86]. If BRCA1 is damaged by a mutation, damaged DNA is not repaired properly, and this increases the risk for breast cancer. Many different types of mutations in BRCA1 have been identified and some of these are harmful, while others area benign. Harmful mutations may result in a hereditary breast-ovarian cancer syndrome. High-risk mutations are those which disable the error-free DNA repair process of homology-directed DNA repair and increase the chance of cancer. The large majority mutations in the BRCA1 genes that have been identified are point mutations and small insertions/deletions. [87]. Thus, it should be possible to use the CRISPR/Cas9 system as a corrective method, although efficient delivery is a major challenge.

As noted above for oncogenes, viruses can cause some human cancers through the elaboration of viral proteins and it is also the case that some human cancer viruses express proteins that interfere with tumor suppressor function. Human papillomaviruses HPV16 and HPV18: Human papillomaviruses (HPVs) are an established etiological agent of human cancer and the high risk or oncogenic human papillomaviruses, HPV16 and HPV18, are responsible for the majority of HPV-associated cancers [9]. HPV has a circular double-stranded DNA genome of 7-8 Kbp [88] and is the major cause of cervical carcinoma and is also involved in other anogenital cancers [9]. The oncogenic properties of high-risk HPVs (HPV16 and HPV18) are conferred by the viral proteins E6 and E7 [89, 90]. E6 targets cellular p53, which is a major cellular tumor-suppressor protein and binds and degrades p53 [91] while E7 complexes with protein members of the retinoblastoma family of tumor suppressors, which control the cell cycle, i.e., pRb, p107 and p130 and this results in their phosphorylation and the release of E2F transcription factors that promote progression of the cell cycle [92]. Thus the E6 and E7 genes are prime targets for CRISPR/Cas9 intervention in HPV-associated malignant diseases. Introduction of Cas9 and E6- or E7-specific gRNAs into HeLa and SiHa cervical carcinoma cell lines which contain integrated HPV18 or HPV16 respectively resulted in inactivating deletion and insertion mutations being introduced into E6 or E7 leading to reactivation of p53 or pRb [93]. This produced cell cycle arrest and subsequently cell death. Hu et al [94] reported that CRISPR/Cas9 with an HPV16-E7-specific gRNA disrupt HPV16-E7 DNA at specific sites and this resulted in apoptosis and growth inhibition in HPV-positive SiHa and CaSki cells, but not in HPV negative C33A and HEK293 cells. CRISPR/Cas9 targeting the promoter or transcription units of HPV16 E6/E7 in SiHa cells resulted in accumulation of p53 and p21 proteins and reduced cell proliferation and tumorigenesis in nude mice [95]. Thus CRISPR/Cas9 is effective in cultured HPV-transformed cell lines and may have potential as an effective therapy for HPV-associated clinical tumors.

**Table 2:** Strategies for CRISPR/Cas9 interventions targeting viral cellular genes in cancer

Gene type	Virus	Family	Gene	Function	Approach	Reference
*Oncogene*	Hepatitis B	Hepadna-virus	Various		Disruption	62-64, 68, 70
			S	Surface antigen	Disruption	65, 67
				Signal transduction	Disruption	65, 67
			Core, P,	Viral capsid	Disruption	69
				DNA polymerase	Disruption	69
			X	Signal transduction	Disruption	69
	Epstein-Barr	Herpes-virus	Various		Disruption	75
			BART	MicroRNAs	Disruption	76
*Tumor Suppressor Inactivators*	HPV16/18	Papilloma-virus	E6	p53 inactivator	Disruption	93, 95
			E7	pRb Inactivator	Disruption	93-95
	JCPyV	Polyoma-virus	T-antigen	p53 and pRb inactivator	Disruption	30
	Hepatitis B	Hepadna-virus	HBx	p53 inactivator	Disruption	65, 67, 69

Other human cancer-causing DNA viruses have been reported to express proteins that target tumor suppressors and are suitable for targeting by CRISPR/Cas9. The HBx of HBV has been reported to bind to p53 [96] and CRISPR/Cas9 targeting HBV is discussed above. Merkel cell polyomavirus (MCV) is a double stranded human cancer-causing DNA virus that expresses oncogenic large-T antigen, which targets the p53 and pRb tumor suppressors [97] and is a potential target gene for CRISPR/Cas9. Although, there are no reports of MCV targeting by CRISPR/Cas9, we have successfully targeted the large-T antigen of human polyomavirus JC (JCPyV), which is suspected but not proven to cause brain and other tumors [30].

As well as viral tumor suppressor inactivating proteins, tumor suppressors can also become inactivated by mutations in their cellular genes and these have a huge potential as targets for treating human cancer by specifically correcting them with CRISPR/Cas9. Tumor suppressor gene changes occur in many cancers and are maybe even more important for malignant cell development than oncogene mutations. CRISPR/Cas9 could be targeted against the mutated form of the tumor suppressor gene but this will be more challenging than oncogene targeting where disruption is the desired effect. Rather precise and efficient gene correction is required. However, CRISPR/Cas9 has the potential to be developed into a specific and efficacious approach to correct these types of changes in cancer cells.

### Epigenetic factors and control loci

The control of gene expression by epigenetic regulators is often dysregulated in cancer cells and such changes are necessary for the process of carcinogenesis [98]. DNA methyltransferases (DNMTs) and enzymes involved in histone modifications, e.g., LSD1, EZH2 and NSD2, are often altered in malignant cells and there is evidence that these epigenetic changes are essential for the maintenance of tumors. Thus, targeting epigenetic regulatory enzymes might be a potential approach for cancer therapy as has been suggested recently for the histone deacetylase family of proteins [99]. One mechanism whereby tumor suppressors can become silenced is gene-specific hypermethylation, which is frequently found in the promoters of genes such as p53, PTEN, BRCA1, etc. [100]. The maintenance of this DNA methylation requires the activity of the DNMTs raising the possibility that DNMTs may be a suitable target for cancer therapy [101]. Similarly, changes in the pattern of histone modifications have been detected in cancer, e.g., the histone acetylation and dimethylation of the five residues in histones H3 and H4 in prostate cancer [102]. Several of the enzymes that are responsible for histone modifications have been found to be mutated in human cancers, e.g., EZH2 [103] and LSD1 [104]. On the basis of such findings, epigenetic enzymes are attractive targets for cancer therapy. In this regard, histone deacetylase inhibitors and DNMT inhibitors have been demonstrated to inhibit the growth of cancer cells. However, the limited specificity of such inhibitors mean that improved strategies of targeting need to be developed [98]. Thus, it is attractive to design CRISPR/Cas9 approaches to epigenetic regulators.

### Chemoresistance genes

The ability of cancer cells to develop chemoresistance to drugs is a major obstacle in many cancer therapies. The principal mechanism by which many cancers develop resistance to chemotherapy drugs is multidrug resistance, which is a major factor in the failure of many forms of chemotherapy [105]. Chemotherapy applies a selective pressure to tumor cells leading to the emergence of drug-resistant cells and resulting in the failure of treatment. At least two types of plasma membrane molecular pumps have been implicated that actively expel chemotherapy drugs from the tumor cells. The first of these to be discovered was P-glycoprotein, a glycoprotein of 170 kD, the expression of which correlated with the degree of drug resistance in several cell lines and mediates resistance to drugs such as colchicine, vinblastine, doxorubicin, etoposide, taxol and others [106]. The gene for P-glycoprotein is MDR-1, which was cloned in 1985, and encodes an energy-dependent pump that expels small molecules from inside cells [107]. A second pump protein, MRP, was cloned in 1992 [108], and both MRP and P-glycoprotein are significant targets for anticancer compounds and would be highly suitable targets for CRISPR/Cas9 therapy.

Enzymes that either activate or inactivate chemotherapeutic drugs are also important in the chemoresistance of some cancers [11]. Mechanisms that inactivate drugs can reduce the amount of drug available to its cellular target. For example, platinum drugs such as cisplatin and oxaliplatin can form conjugates with glutathione (GSH) resulting in their inactivation of these drugs [109]. Anticancer platinum drugs become covalently linked to GSH and subsequently efflux via ABC transporter proteins [110]. GSH conjugation is catalyzed by the glutathione-S-transferase (GST) enzyme family, with elevated levels of expression of the GST-p subgroup being associated with cisplatin resistance in ovarian cancer cells [111]. Another example is irinotecan, which is a substrate for inactivation by cytochrome P450 enzymes and SN-38, which is a target for glucuronidation by uridine diphosphogluronysl transferase 1A1 (UGT1A1)[112, 113]. Inactivation of a gene that encodes an enzyme that is important in the chemoresistance of a given cancer using CRISPR/Cas9 is a potential treatment that could be given prior to or in conjunction with chemotherapy.

## DELIVERY OF CRISPR/CAS9

Perhaps the greatest challenge ahead is the efficient delivery of CRISPR/Cas9 to the targeted cancer cells. A number of approaches are possible including viral transduction using adenovirus, adeno-associated virus (AAV) or lentiviruses [114–116] and nonviral physical methods [117]. The usefulness of adenovirus vectors is limited by their immunogenicity [114]. Lentiviral vectors, which are often based on HIV-1, make permanent genetic changes in the target cells since they have integrase-dependent mechanisms of semi-random integration into the host genome. However, for CRISPR/Cas9 editing unlike in classic gene therapy approaches, it is important that the presence of the transduced nuclease be transient to limit off-target events and integrase-defective lentiviruses (IDLV) are preferable [115]. Self-inactivating lentiviruses replication-incompetent lentiviruses also give transient expression and have the ability to transduce both dividing and nondividing cells [116]. The feasibility of lentiviral CRISPR/Cas9 delivery vectors is illustrated by many studies including the eradication of latent infection by HIV-1 [28], HBV [63, 66, 68] and herpervirus [75]. Unlike lentiviruses, AAVs lack an integration machinery and so their genomes remain mostly in an episomal state. Recombinant AAV vectors have a low pathogenicity, low immunogenicity capability to transduce both dividing and nondividing cells and do not integrate. One of the limitations of AAV is the size limitation of the transgene. Swiech et al [118] developed a system to deliver Cas9 and gRNAs in separate AAV vectors. Another possibility is to split the Cas9 enzyme in halves that are delivered separately. Wright et al [46] designed a split-Cas9 enzyme where the nuclease lobe of Cas9 and the α-helical lobe of Cas9 are expressed as separate polypeptides. The sgRNA recruits the halves into a functional ternary complex which the activity of full-length Cas9 and catalyzes site-specific DNA cleavage [46]. Similarly, Truong et al [47] developed a split-Cas9 using split-inteins and intein-mediated trans-splicing reconstituted the full-length Cas9 protein. Ran et al [45] found that Cas9 from Staphylococcus aureus (SaCas9) can edit the genome with efficiencies similar to those of the usual Cas9 from Streptococcus pyogenes, while being more than 1 kilobase shorter [45]. A final possibility is the development of nonviral delivery methods but these have been mainly limited to in vitro systems [117].

## CONCLUSIONS AND PROSPECTS

CRISPR/Cas9 gene editing is a relatively recent technology that has a huge potential for making highly specific genetic changes in cellular DNA to treat a variety of diseases including cancer. Many obstacles need to be overcome, especially in the area of gene delivery and reduction of off-target effects but this system holds huge potential in the area of cancer therapy.
